# A comparison of visual attention to pictures in the Autism Diagnostic Observation Schedule in children and adolescents with ADHD and/or autism

**DOI:** 10.3389/fpsyt.2024.1378593

**Published:** 2024-04-29

**Authors:** Puja Kochhar, Iti Arora, Alessio Bellato, Danielle Ropar, Chris Hollis, Madeleine (Maddie) J. Groom

**Affiliations:** ^1^Neurodevelopmental Specialist Service, Nottinghamshire Healthcare National Health Service (NHS) Foundation Trust, Nottingham, United Kingdom; ^2^Institute of Mental Health, University of Nottingham, Nottingham, United Kingdom; ^3^Division of Psychology and Language Sciences, Faculty of Brain Sciences, University College London, London, United Kingdom; ^4^School of Psychology, Faculty of Environmental and Life Sciences, University of Southampton, Southampton, United Kingdom; ^5^School of Psychology, University of Nottingham, Nottingham, United Kingdom; ^6^National Institute for Health and Care Research (NIHR) MindTech Medtech Co-operative, Institute of Mental Health, UK NIHR, Nottingham, United Kingdom; ^7^Nottingham Biomedical Research Centre, Institute of Mental Health, Nottingham, United Kingdom

**Keywords:** Attention Deficit Hyperactivity Disorder, autism, autistic spectrum disorder, Autism Diagnostic Observation Schedule, eye-tracking, comorbidity, diagnosis

## Abstract

**Background:**

Attention-Deficit/Hyperactivity Disorder (ADHD) and Autism Spectrum Disorder (ASD) are neurodevelopmental conditions which frequently co-occur. The Autism Diagnostic Observation Schedule (ADOS) is commonly used to aid with diagnostic assessment of ASD but was not originally designed for use in those with comorbid ADHD. Visual attention to social stimuli has been often studied in ASD using eye-tracking, to obtain quantitative indices of how attention is deployed to different parts of a social image/scene. As the ADOS includes tasks that rely on attending to and processing images of social scenes, these measures of visual attention could provide useful additional objective measurement alongside ADOS scores to enhance the characterisation of autistic symptoms in those with ADHD.

**Methods:**

Children with ASD, comorbid ASD and ADHD, ADHD and Neurotypical (NT) controls were recruited (n=84). Visual attention was measured using eye-tracking during free viewing of social scenes selected from the ADOS. The full ADOS was then administered. Stimulant medication was temporarily withdrawn during this assessment. Research diagnoses were based on the Development and Wellbeing Assessment (DAWBA), ADOS, Social Communication Questionnaire (SCQ, a measure of ASD severity) and Conners’ Rating Scales (CRS-3, a measure of ADHD severity) following clinical consensus.

**Results:**

Using factorial ANOVAs to model ADHD, Autism and their interaction, we found that fixation duration to faces was reduced in those with ASD (ASD and ASD+ADHD) compared to those without ASD (ADHD and NT). Reduced visual attention to faces in the whole sample was associated with Autism symptom severity (SCQ subscale scores) but not ADHD symptom severity (CRS-3 scores).

**Discussion:**

Our findings provide preliminary evidence in support of implementing visual attention measurement during assessment of ASD in the context of comorbidity with ADHD. For example, if a child with ADHD was found to reduce attention to faces in ADOS pictures this may suggest additive difficulties on the autism spectrum. Replication across a larger sample would be informative. This work has future potential in the clinic to help with complex cases, including those with co-occurring ADHD and ASD.

## 1 Introduction

Attention-Deficit/Hyperactivity Disorder (ADHD) is a common heterogeneous neuro-developmental condition characterised by developmentally inappropriate levels of hyperactivity, impulsivity and inattention (1). Autism Spectrum Disorder (ASD) encompasses impairing reciprocal social communication difficulties in addition to restrictive repetitive behaviours (1). Although ICD-10 and DSM-IV stipulated that children referred for ADHD diagnostic assessment should not meet the criteria for ASD; DSM-5 now allows for the dual diagnosis of both ADHD and ASD (1–3).

Current diagnostic methods are generally based on assessing for individual diagnoses separately even though cooccurrence is common (4, 5). The consequence is that children with ADHD who have significant social-emotional difficulties (including ASD), which cause impairment, are often missed at the outset (6). This can lead to extensive morbidity for the individual and poor socio-economic outcomes for society as it has been shown that children with ADHD who show increased emotional dysregulation and social dysfunction, have a poorer prognosis (7–9).

Previously, socio-emotional difficulties in ADHD have been assumed to be a consequence of core ADHD symptoms such as inattention, poor listening skills, difficulties waiting their turn and social impulsivity (10). However, the fact that social-emotional problems persist in many individuals treated with stimulant medication that are so effective at improving core ADHD symptoms (11–13), suggests that other mechanisms could be leading to socio-emotional difficulties in ADHD. The comorbidity between ADHD and ASD could be one possible explanation for the socio-emotional difficulties in ADHD. There is, however, still uncertainty whether all socio-emotional difficulties are due to an independent ASD diagnosis, diagnostically subthreshold ASD symptoms, or a severe and broader ADHD phenotype; therefore, it is important to understand this more fully.

In research and clinical practice, the Autism Diagnostic Observation Schedule (ADOS) is used in the assessment of ASD and includes tasks designed to identify emotion recognition and to test for processing and comprehension of social information. The ADOS was designed to be used in community populations to distinguish ASD from typically developing children. It’s validity to assess ASD in those with ADHD is unclear (14). For example, in some children with both diagnoses of ADHD and ASD, scores on the ADOS can be within the range of those with an ADHD diagnosis alone (15). In those with ADHD without a diagnosis of ASD, scores on the ADOS can be raised above the threshold for ASD across the lifespan (10, 16, 17). Furthermore, in verbal adolescents, the specificity of the ADOS has been shown to be low for ASD versus those without ASD (including ADHD) (18). Overall, these studies suggest that ASD diagnoses maybe misdiagnosed or missed in those with ADHD. Additionally, although the ADOS is an observer-based tool by a trained clinician, it can still be prone to subjective bias by the administrator.

Given the high rates of comorbidity between ADHD and ASD, and the evidence of socio-emotional difficulties in ADHD, it is important to objectively clarify the use of the ADOS in children and adolescents with ADHD. In particular, as children with ADHD may have independent difficulties in socio-emotional functioning due to their ADHD symptoms, the ADOS scores could be artificially raised leading to a false positive diagnosis of ASD on this instrument (15). Alternatively, the ADOS may be well-placed to detect co-occurring ASD symptoms in those with ADHD, supporting a dual diagnosis when appropriate, and providing valuable information for treatment. Further research is needed to investigate whether the ADOS is sensitive to ASD features in children who have both ADHD and autism, and whether performance is also influenced by core symptoms of ADHD.

Visual attention, often assessed using eye-tracking to obtain quantitative indices of how attention is deployed to social stimuli, has been extensively reviewed in the ASD literature. The most consistent finding is that children with ASD process social information differently than those without ASD; for example when assessing visual attention using eye tracking, the time spent to focus on the social areas/components of certain scenes (such as the eyes and face) was found to be reduced in ASD compared to neurotypical controls (19–23). Furthermore, visual attention has been found to be more altered in ASD when the scene is more complex (24) and, orienting to faces in social scenes has been shown to be slower in ASD compared to neurotypical controls (25) and those with ADHD (26). Visual attention to social stimuli in children with ADHD has been found to be slightly reduced or similar to neurotypical children; these findings may be explained depending on the emotion depicted in the scene for example, Serrano et al. (2018) found that children with ADHD had highest effect sizes for reduced visual attention to angry/scared faces however effect sizes for reduced visual attention to happy/neutral faces were modest compared to neurotypical children (27).

### 1.1 Aims & hypotheses

The main aim of this study is to compare visual attention to ADOS pictures amongst children and adolescents with ADHD, ASD, ADHD+ASD, and Neurotypicals. As the ADOS includes tasks that rely on attending to and processing social scenes, eye-tracking can provide quantitative and objective measures of visual attention to the scenes, alongside ADOS scores.

To quantify visual attention to ADOS pictures, eye tracking measures commonly used in the ASD literature described above were derived. Viewing time, otherwise known as fixation duration or Dwell Time (DT), is a measure of how long an area of interest (e.g., a face) is looked at when an individual is presented with a social scene. DT taps into attention duration. The number of times an area in the social scene is looked at is measured by the Fixation Count (FC), and how long it takes to look at an area of interest (orienting) is measured by the First Fixation Time (FFT). Measures of visual attention to Interest Areas, including social areas (faces) and non-social areas (vehicles/buildings), were compared between the groups, in the present study.

Based on the literature presented above, it was hypothesised that children with ASD would have reduced viewing time/interest (DT), exploration (FC) and slower orienting (FFT) to the social areas (faces) than the non-social areas of the pictures, compared to neurotypical children and children with ADHD. It was also hypothesised that this profile of atypical visual attention would be more pronounced for the pictures with highest content density.

It was hypothesised that, in neurotypical controls, visual attention to social areas (faces) would be greater than non-social areas (vehicles/buildings). The predicted profile would include increased DT (indexing viewing time/interest), increased FC (indexing exploration) and, quicker FFT (indexing faster orienting) to social areas (faces) compared with non-social areas in the pictures.

We predicted that the ADHD group would show less impaired attention to social parts of the ADOS pictures (happy and neural emotional content) compared to the ASD group and would be more like the neurotypical controls. It was postulated that the ADHD group may still favour social over non-social areas if their visual attention is impacted by a general impairment in attention rather than socio-emotional difficulties per se. This would manifest in slightly reduced DT and FC, and slower FFT to non-social parts of the image compared to neurotypical controls. In those with both co-occurring ASD and ADHD, we did not specify one-tailed predictions due to the relative lack of prior literature on eye-tracking in this population. However, we reasoned that if atypical attention to the ADOS images is driven by the presence of ASD symptoms, they would show a profile more like the ASD group, reflected in reduced DT, FC and longer FFT to the social areas specifically when compared with non-social areas. Conversely, if atypical attention was primary due to ADHD symptoms, the comorbid group may show a profile of predominantly reduced DT, FC and longer FFT to non-social areas. To test our hypotheses, we modelled ADHD and ASD as between-subjects factors and tested the main effects of each on DT, FC and FFT, and the interaction with the type of interest area (social, non-social). We also tested the interaction between ADHD and ASD factors, to determine whether the comorbid group showed a unique pattern when compared with either the ADHD or ASD groups.

## 2 Methods

### 2.1 Participants

Participants were recruited as part of a larger study called the Study of Attention and Arousal in Neurodevelopmental Disorders—SAAND, funded by The Baily Thomas Charitable Fund and The Waterloo Foundation (grant number 980-365) within the Division of Psychiatry and Applied Psychology at the University of Nottingham. Ethical approval for the study was given by the East Midlands Research Ethics Committee (REC) (17/EM/0193), IRAS project number 220158. A large proportion of the children recruited to the SAAND study also took part in this eye tracking study. Detailed methods and results from the SAAND study are reported elsewhere (24, 28, 29).

#### 2.1.1 Clinical groups

Children and adolescents aged 7–15 years with a clinical diagnosis of ADHD, ASD or ASD+ADHD were recruited from Child and Adolescent Mental Health Services (CAMHS) and community paediatric clinics in Nottinghamshire. Some children were also recruited from local ASD and ADHD charities. Children from ASD and ADHD support groups were also informed of the study by the support group convenor. Although many children had prior diagnoses of ASD or ADHD, some children (awaiting assessment) who were deemed high risk by CAMHS of a neurodevelopmental disorder were recruited as long as they met study inclusion criteria for the SAAND study (24, 28, 29). After referral to the research study, a full research diagnostic assessment was undertaken on each clinical case using the measures described in section 2.2 below. This was to ensure correct assignment to one of 3 clinical groups: ADHD, Autism, comorbid ADHD and autism.

The initial clinical diagnoses were confirmed or overturned by PK (an experienced clinical rater) after the Development and Wellbeing Assessment (DAWBA) transcripts (30) and screening questionnaires were completed. Further details of how DAWBA diagnoses were operationalised are given below. Where diagnostic decisions were complex, clinical consensus with at least two child and adolescent psychiatrists (PK and CH) took place. This included discussing all the information available and assigning the final diagnoses based on the overall consensus. This methodology has been employed previously by PK and CH (31, 32).

Children with comorbid diagnosis of epilepsy or learning disability (IQ<70), and children with visual problems (such as colour blindness) and hearing problems, were excluded. Children with comorbid mental health conditions including anxiety disorders, mood disorders and conduct disorders were included in recognition of the prevalence of these comorbidities with ADHD and autism. Children with comorbid specific learning disorders, e.g., dyslexia or developmental coordination disorder, were also included.

This process resulted in 16 children assigned to the ADHD group, 18 assigned to the Autism group, and 28 to the comorbid group.

Patients recruited to the study and taking stimulant medication were asked not to take this medication on the day of the study. Patients taking ADHD non-stimulant medication such as atomoxetine were excluded due to its longer duration of action and those taking dexamphetamine were also excluded due to its low rate of prescribing. Children on medication for sleep such as melatonin or taking Selective Serotonin Reuptake Inhibitors (SSRIs) were also included. Children taking antipsychotics such as risperidone were excluded as this could potentially affect our measures of interest and not be easily withdrawn.

#### 2.1.2 Neurotypical control group

Letters detailing the study were sent to families of children in primary and secondary schools in the Nottinghamshire region. From an initial sample who volunteered to take part, a group of controls matched pairwise for age (± 6 months) to individuals in one of the clinical groups were selected. Eligibility for the neurotypical control group was determined by asking parents to complete the Conners parent rating scale and Social Communication Questionnaire. Those with significant ADHD or ASD symptoms on the Conners (T score > 65) or SCQ (total score > 15) were excluded from the study.

### 2.2 Measures

To establish assignment to one of the groups, a combination of questionnaires, interview and observational measures were used. Children were screened for ASD using the SCQ-lifetime version (33), a 40-item, parent-rated questionnaire which provides an overall index of risk of autism spectrum condition. The scale also provides scores relevant to 3 sub-scales: social reciprocal interaction, communication, and repetitive stereotyped behaviours. A score of 15 on the SCQ is a recognised, evidence-based cut-off for differentiating those at risk of ASD from neurotypical children (34).

Parents and teachers also completed the Conners’ Rating Scales (CR-3) (35). To be included in the ADHD group or comorbid ADHD+ASD group, the T-scores on the Conners’ Rating Scales had to be more than 1.5 standard deviations above the mean on the attention scale (this equates to T-scores of more than 65). Parents completed the Strength and Difficulties Questionnaire (SDQ) (36). The SDQ is a 25-item questionnaire with a 3-point scale (not true, somewhat true and certainly true) to measure emotional and behavioural difficulties in children. Five domains are measured (conduct, emotions, hyperactivity, peer problems and prosocial behaviour), each using 5 questions. Participants required a parent reported hyperactivity score of more than 5 to be included in the ADHD group or comorbid ADHD+ASD group.

In the clinical groups, all scales were rated when the child was off medication and the parent was asked to rate what their child was like when they were off medication in the last six months; however, the SCQ also required parents to comment on their child’s behaviour when they were aged 4–5 years old.

The Development and Wellbeing Assessment (DAWBA) is a structured interview, which can be administered by interviewers to informants or completed directly online by parents, teachers or adolescents (11–17 years old) to generate DSM-IV and ICD-10 diagnoses. In this study, the DAWBA was administered online to parents. The DAWBA measures emotional, behavioural and hyperactive disorders and also has a developmental section covering ASD (30). Children’s parents completed the Social Aptitudes Scale (SAS) as part of the Development and Wellbeing Assessment (DAWBA) (30). The SAS is a 10-item scale and is a broad measure of complex interactive social skills. Parents are asked to compare their child’s behaviour across a range of situations to their peers. Scores can range from 0 to 40 and low scores have been shown to be associated with ASD in community samples (37). It should be noted that higher rates of difficulties on the SAS than the SCQ have been found in those with ADHD without a comorbid diagnosis of ASD in a clinical sample (38). A score of 12 or less on the SAS indicates difficulties in social functioning and necessitates that all items of the development Section (ASD diagnostic Section) of the DAWBA are completed.

All clinical participants were invited to attend an Autism Diagnostic Observation Schedule 2^nd^ Edition (ADOS-2) (39) as part of the research diagnostic assessment. ADOS-2 assessments were carried out by PK and IA, who have research reliability accreditation. Module 3 or 4 of the ADOS was used depending on chronological age and verbal fluency of the participant. The assessment takes 45 minutes to complete, and the child is observed carrying out 14 tasks (e.g. description of a picture). The assessment provides scores in the domains of communication, reciprocal and social interaction, and stereotyped behaviours and restricted interests. Based on cut-off scores, an ADOS classification of autism or autism spectrum is generated, which is used to help reach a research diagnosis in the clinical consensus. Within the reciprocal social interaction domain, the ADOS also has scores related to emotion recognition. For the purposes of this study, the ‘Comments on Others’ Emotions/Empathy’ subscore was used in further analysis. This subscore reflects the participants’ spontaneous emotion recognition, understanding and response to feelings of others throughout a series of tasks. It was predicted that this particular subscore would be closely related to visual attention to faces within the ADOS pictures. It is scored from 0 to 2 with higher scores reflecting less spontaneous emotion recognition. Further details of how this ADOS emotion recognition subscore is generated in the ADOS scoring procedure, are presented in the **Supplementary Materials**.

To screen for learning disability, all children completed an abbreviated intelligence test, Wechsler Abbreviated Scale of Intelligence (WASI) (40), which is an abbreviated measure of IQ and takes around 20 minutes to complete. Two sections (vocabulary and block design) enable performance, verbal and full-scale IQ to be generated. Any participants with a full-scale IQ of less than 70 were excluded from the study.

### 2.3 Eye tracking procedure and task stimuli

To measure visual attention to social scenes, participants viewed stimuli from the ADOS Description of a Picture Task while their eye gaze was measured on the EyeLink 1000 Plus eye tracker (SR Research Ltd. 2017) at a sampling rate was 500Hz (sampling every 2ms). Participants’ rested their chin on a chin-rest to reduce head movement and maximise comfort during the procedure. Stimuli were presented on a screen (48cm wide x 27cm height), 60cm from the participant. A 9-point calibration was completed prior to the task. The laboratory eye tracking room had no natural light allowing the lighting in the room to be kept constant during the eye tracking procedure. Laboratory room luminance was measured using a photometer to ensure luminance consistency (between 70–90 lux) for each testing session. ADOS pictures were resized to 16cm wide x 10cm height. A central fixation cross was presented for 100ms interspersed by the ADOS pictures which were presented in a random order for 20 seconds each. Participants were verbally instructed to look at the stimuli however they liked.

Stimuli consisted of the three pictures selected from the ADOS ‘Description of a Picture’ task. These pictures depict scenes of a Holiday, of Hollywood and of people eating a Meal (39). The pictures from the ADOS are colourful, content dense with many faces and other objects such as vehicles or planes that can take the participant’s interest. The faces were classified as social interest areas while the vehicles and buildings were classified as non-social interest areas. The majority of the faces in the pictures range from neutral to positive valence emotions. The 3 pictures range slightly in content density with the highest number of faces in the Holiday picture, the least number of faces in the Meal picture and the Hollywood picture having a number midway, between the other two pictures. The crowding of the components and the contrast of colours was also deemed to be in a similar descending order for the 3 pictures (with the Holiday picture having the most components and brightest colours and the Meal the least). The valence and content density of the pictures was defined in this way for analysis.

For the ADOS pictures, two types of interest areas were created a priori, faces (social areas) and non-social areas. Faces needed to be in full view to be selected as a social interest area (for example the back of the head or faces obscured by a large hat or glasses were not selected). Non-social areas included any vehicle or building in the pictures and were selected as they were deemed as the most non-social ‘mechanical’ areas of the pictures. Although food and trees could be classed as non-social, these were not included as they are more organic in nature and could be visualised for other reasons for example food due to hunger in the participant. As the ADOS pictures are copyrighted these cannot be displayed in the manuscript however the reader is referred to the ADOS-2 manual for further detail (39).

The eye tracking tasks to the ADOS pictures was completed before the full ADOS assessment which was administered in full separately. The eye tracking tasks, including the calibration took approximately 5 minutes for participants to complete. Raw eye tracking data was processed using the EyeLink 1000 Plus accompanying software EyeLink Data Viewer. This software allows for interest areas such as faces to be isolated from the rest of the image and visual attention to be measured within the specified interest area. In addition to Dwell Time which is an indication of viewing time to the specified interest area, this software provides automated extraction of a wide range of additional eye tracking measures defined within the specified interest area.

Total stimulus length was analysed which corresponds to 20 seconds for the ADOS images. A minimum fixation duration of 100ms was set to allow for shorter fixations while scanning complex pictures as opposed to faces only (41). Blink artefacts were removed by defining these as any periods of data where pupil size was equal to 0mm.

Trials were removed if eye movement data was not continuously recorded for at least 25% of the trial time. Trial adequacy was verified using a two-step approach; firstly, the data was visually assessed in Data Viewer and secondly, a percentage acquisition was computed by Data Viewer for cross checking. Participants with more than 50% invalid trial data were removed from the analysis, as has previously been documented in the literature with similar trial numbers in ASD and disruptive disorders (42). Of the initial 87 eligible children recruited, two control children and one child in the ASD+ADHD group did not have adequate eye movement data using the described procedure so 84 children were included in the final eye movement data analysis.

As the stimuli vary based on colour, emotions and density, it can be conceived that using one single visual attention measure may fail to disentangle what might be driving viewing patterns to these stimuli. A range of eye tracking measures are likely to be required to distinguish complex neurodevelopmental disorders and, attentional patterns and priorities to people in scenes have been analysed by multiple eye tracking measures in ASD previously (25). Eye tracking measures from social scene perception literature in ASD were included for extraction in this study as described below. In addition to Dwell Time (DT) which is an indication of viewing time to the specified interest area, two additional eye tracking measures were chosen for the main analysis. These included the First Fixation Time (FFT) and the Fixation Count (FC). The FFT to an interest area is indicative of orientation to an interest area, for example time to orient to a face. This was important to test as faces are not preselected in everyday life and those with ASD have been shown to have slower orientation to faces (25, 26). The FC which is a measure of the number of fixations within an interest area was also extracted. The FC allows for the estimation of the exploration and processing of a face.

The pre-selected eye tracking measures described above were extracted using the interest area report function in EyeLink. For the ADOS pictures, the sum of all the variables was calculated, except the FFT which was the minimum value.

### 2.4 Statistical analysis

Group differences for the demographic and clinical scores were calculated using univariate ANOVAs and followed up using *post hoc* tests adjusted for multiple comparisons. If Levene’s test of difference between group variances was significant, the Games-Howell *post hoc* test was used; otherwise, Tukey’s test was used.

A series of ANOVAs were performed to test the main effects of, and interactions between, two between-subject factors ASD (yes, no) and ADHD (yes, no) on eye-tracking variables (DT, FC and FFT). ASD factor (yes) includes children from the ASD group and ASD+ADHD group. ASD (no) includes children from the ADHD group and control group. ADHD factor (yes) includes children from the ADHD and ASD+ADHD group and ADHD (no) includes children from the ASD and control groups.

Within-subjects factors Interest Area, comprising two levels (Social, Non-social), and Picture, comprising 3 levels (Meal, Hollywood, Holiday), were entered. The Picture factor was manipulated in this way to reflect increasing image content density (defined by the number of faces, the crowding of the components and the contrast of colours in the ADOS pictures) as it was hypothesised that visual attention allocation would differ based on content density. Significant interactions were followed up by simple effects analysis (pairwise comparisons). As DT percentage gave a similar pattern of results to DT, only results for DT are reported for brevity.

The two factor design tests the main effect of ASD and ADHD factors on variables of interest in addition to an interaction between ASD and ADHD factors (43, 44). In particular, an interaction between ASD and ADHD factors could suggest a model compatible with the comorbid group belonging to a third separate nosology to either of the pure groups as described by Rutter and Taylor (2002) (45). Alternatively, a main effect of ASD and/or ADHD factors, with a lack of interaction between ASD and ADHD factors could suggest an ‘additive model’ of comorbidity, especially if a double dissociation exists (43). This additive model would be in keeping with the comorbid group sharing a profile with both the pure groups and potentially shared risk factors within the comorbid group.

Pearson’s correlation analyses were conducted to analyse associations between symptom severity subscales (SCQ and CRS-3) including the ADOS emotion recognition subscore and the eye tracking variables that were found to be predictors of ASD or ADHD factors in the factorial ANOVAs described above.

To examine the possible effects of covariates, hierarchical linear regression was used to test if FSIQ or oppositional symptoms (Conners’ parent rating scale oppositional subscale T scores) had significant contributions to the dependent variable over and above ADHD and ASD factors. Hierarchical linear regression models were applied to the dependent visual attention variable being tested (e.g., DT to ADOS pictures). This analysis was performed only on dependent variables that were found to be significant in the main analyses described above. Further details of the model design and the results of the analysis are presented in the **Supplementary Materials**.

## 3 Results

As shown in **Table 1**, groups were well matched for age and there was a male predominance in all groups. Groups differed in mean Full Scale IQ (*F* (3, 79)=5.66, p<0.01, ηp^2^ = 0.18) with significantly lower scores in the ASD group (p<0.01) and ASD+ADHD group (p<0.01) compared to the control group.

As expected, groups differed significantly on the Conners’ DSM-5 subscale T-scores with significantly higher T-scores in all three clinical groups compared to control group (p<0.001). The groups differed significantly on SCQ (*F* (3, 77)=34.11, p<0.001, ηp^2^ = 0.57) with significantly higher scores in all the clinical groups (p<0.001) compared to the control group. SCQ scores were also significantly higher in the ASD+ADHD group (p<0.05) compared to the ADHD group (**Table 1**). The clinical groups differed significantly on total ADOS scores (*F* (2, 53)=21.76, p<0.001, ηp^2^ = 0.45) with significantly higher scores in the ASD group (p<0.001) and the ASD+ADHD group (p<0.001) compared to the ADHD group. The clinical groups did not differ significantly on the ADOS emotion recognition sub-score (*F* (2, 53)=1.73, p>0.05, ηp^2^ = 0.06) (**Table 1**).

**Table 1:** Demographic and clinical characteristics of the sample (n=84).

	Group				Group effect		Group differences (post-hoc)
	ADHD (n=16)	ASD (n=18)	ASD+ADHD (n=28)	Controls (n=22)	*F*	p	
**Mean age in years** **(SD)**	10.17 (2.06)	11.01 (2.11)	10.82 (1.56)	10.80 (2.40)	0.55	>0.05	n/s
**Gender % male** **(n male)**	68.8% (11)	61.1% (11)	75.0% (21)	59.1% (13)	0.56	>0.05	n/a
**Mean FSIQ** **(SD)**	111.44 (10.37)	103.53 (15.41)	103.04 (19.47)	119.00 (9.83)	5.66	<0.01	Controls > ASD^/^ASD+ADHD ^b^
**Conners’ IA T score** **(SD)**	83.50 (10.97)	77.44 (12.47)	84.04 (7.62)	52.05 (8.81)	52.38	<0.001	Controls < ADHD/ASD/ASD+ADHD ^a^
**Conners’ HI T Score** **(SD)**	85.69 (7.17)	75.71 (12.57)	84.70 (8.92)	53.23 (10.26)	50.74	<0.001	Controls < ADHD/ASD/ASD+ADHD ^a^ ASD < ADHD/ASD+ADHD ^c^
**Conners’ OD T Score** **(SD)**	81.75 (10.55)	77.12 (15.08)	83.48 (11.04)	52.95 (10.27)	32.31	<0.001	Controls < ADHD/ASD/ASD+ADHD ^a^
**SAS** **(SD)**	11.38 (6.75)	7.33 (5.64)	7.42 (5.53)	24.28 (4.87)	37.70	<0.001	Controls > ADHD/ASD/ASD+ADHD ^a^
**Total SCQ** **(SD)**	16.19 (7.22)	19.31 (6.12)	21.41 (6.48)	4.45 (4.75)	34.11	<0.001	Controls < ADHD/ASD^/^ASD+ADHD ^a^ ADHD < ASD+ADHD ^c^
ADOS Total score * (SD)	5.00 (3.35)	14.00 (4.86)	14.08 (5.18)	n/a	21.76	<0.001	ADHD < ASD/ASD+ADHD ^a^
ADOS emotion recognition subscore* (SD)	0.37 (0.50)	0.75 (0.68)	0.67 (0.62)	n/a	1.73	>0.05	n/s
**Oppositional and Conduct Disorders**	50.0% (8)	52.9% (9)	53.6% (15)	0	–	n/a	–
**Emotional Disorders†**	37.5% (6)	64.7% (11)	53.6% (15)	0	–	n/a	–

FSIQ, Full Scale Intelligence Quotient (WASI); Conners’ Parent Rating Scale: IA (Inattentive), HI (Hyperactive-Impulsive), OD (Oppositional) T scores (≥65 suggests difficulties in these areas). SAS, Social Aptitudes Scale (≤ 12 is suggestive of ASD); SCQ, Social Communication Questionnaire (≥15 suggestive of ASD); Rating scale scores shown are parent rated. ADOS, Autism Diagnostic Observation Schedule total score (Autism spectrum cut-off score ≥7). *ADOS total and emotion recognition scores not available for typically developing controls (n=22). †Emotional Disorders include: general anxiety disorder, mild depressive episode, obsessive compulsive disorder or specific phobia (note: participants could have more than one disorder). ^a^p<0.001; ^b^p<0.01; ^c^p<0.05.

Considering first the main effect of Autism on the eye-tracking variables, DT was significantly reduced in those with ASD (1911.10ms, SD=111.02) compared to those without (2245.92ms, SD=117.81; *F* (1, 78)=4.28, p<0.05, ηp^2^ = 0.05). FFT was significantly increased in those with ASD (4952.67ms, SD=399.71) compared to those without ASD (3641.71ms, SD=424.18; *F* (1, 78)=5.06, p<0.05, ηp^2^ = 0.06). There was a trend for reduced FC in those with ASD (6.70, SD=0.04) compared to those without ASD however, this did not reach significance (7.71, SD=0.43; *F* (1, 78)=3.00, p*=*0.09, ηp^2^ = 0.04). There was no significant main effect of ADHD on any of the eye-tracking variables or a significant ASD*ADHD interaction (**Table 2**).

**Table 2:** Summary of main effects of fixed factors (ADHD, ASD, ASD*ADHD) on eye tracking measures in ADOS pictures.

		Main effect (fixed factors)†		
Stimulus	Measure	ADHD	ASD	ASD* ADHD
**ADOS Pictures**	DT	n/s	↓	n/s
	FC	n/s	n/s	n/s
	FFT	n/s	↑ (slower)	n/s

^†^2X2 factorial approach: ADHD (ADHD and ASD+ADHD) versus no ADHD (ASD and controls); ASD (ASD and ASD+ADHD) versus no ASD (ADHD and controls); ASD*ADHD [interaction of fixed factors; comorbid group differs from pure group(s)]. DT, Dwell Time; FC, Fixation Count; FFT, First Fixation Time- ↑(slower)=increased time to fixate and slower to orientate. ADOS, Autism Diagnostic Observation Schedule; Direction of findings are significant (p<0.05); n/s (non-significant; p>0.05).

**Figure 1:**
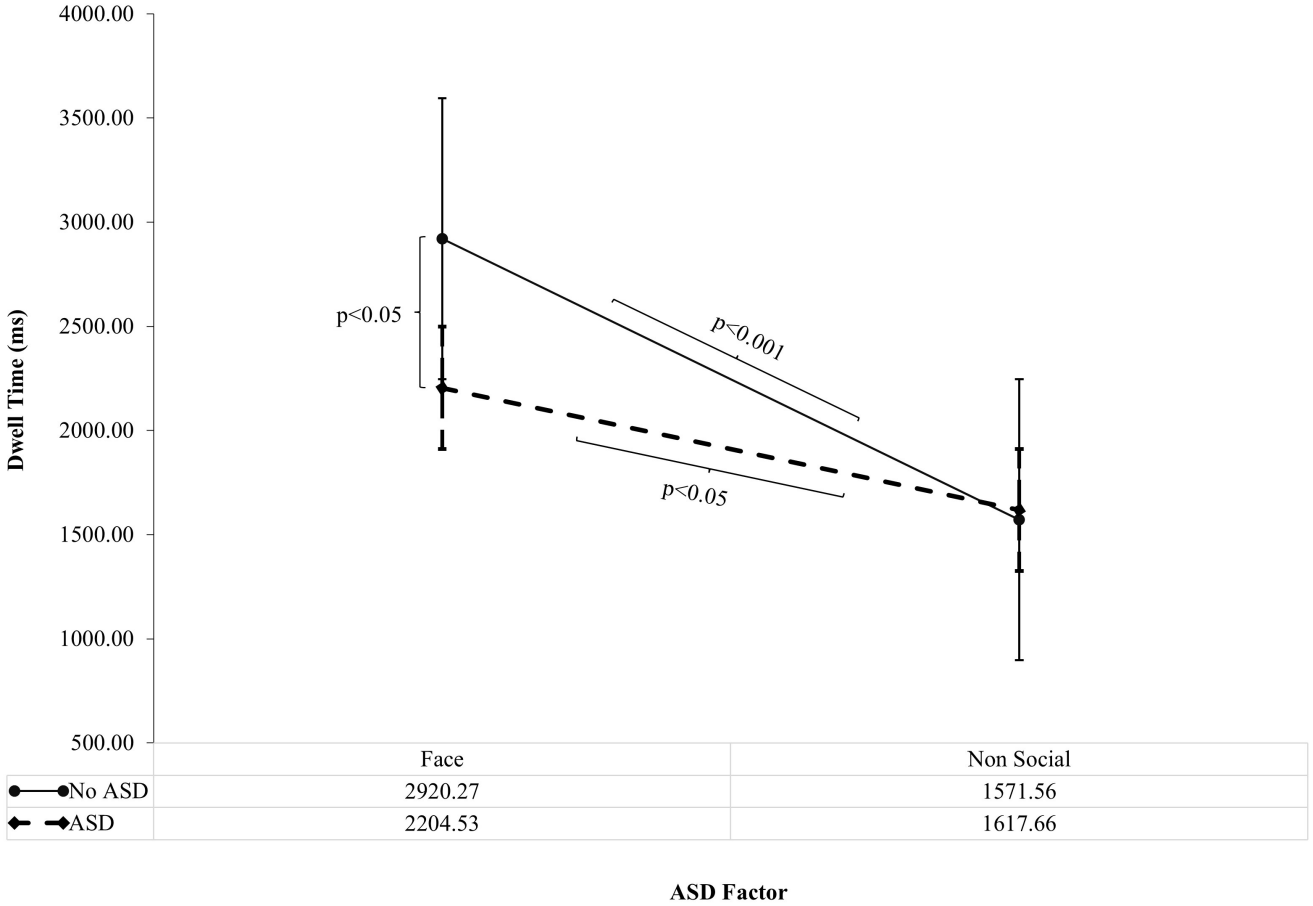
ASD Factor on Dwell Time to Faces versus Non-social areas of ADOS Pictures (Standard Error Bars=95% CI). The interaction between Autism and Interest Area on Dwell Time (*F* (1, 78)=4.16, p<0.05, ηp^2^ = 0.05) was followed up by simple effects analysis. Significance bars denote significant findings from simple effects analysis. ASD = ASD group and ASD +ADHD group; No ASD = ADHD group and Control group.

There was a significant interaction between Autism and Interest Area on DT (*F* (1, 78)=4.16, p<0.05, ηp^2^ = 0.05). The interaction between Interest Area and ASD factor on DT was followed up by simple effects analysis which showed a significantly reduced DT to faces in those with ASD (2204.53ms, SD=210.22) than without ASD (2920.27ms, SD=223.09; *F* (1, 78)=5.45, p<0.05, ηp^2^ = 0.07), as shown in **Figure 1**. There was no significant difference in DT to non-social areas in those with ASD compared to those without ASD. There was significantly increased DT to faces versus non-social areas within ASD (p<0.05) although, this finding was more robust in those without ASD (p<0.001), explaining the 2-way interaction between ASD and Interest Area. Overall, these findings suggest that DT to faces is specifically reduced in those with ASD compared to those without ASD (**Figure 1**).

There was a trend for an interaction between Autism and Interest Area on FC (*F* (1, 78)=3.17, p=0.08, ηp^2^ = 0.04) and FFT (*F* (1, 78)=2.42, p=0.12, ηp^2^ = 0.03) but these did not reach significance. There was no interaction between ADHD factor by Interest Area or by Picture or by ASD factor. Significant multivariate effects of Interest Area, Picture and Picture by Interest Area on all the eye tracking variables that were independent of the fixed factors (ASD factor and ADHD factor) are tabulated in the **Supplementary Material**. Raw eye tracking data in the groups (ASD, ADHD, ADHD +ASD and control groups) are also tabulated in the **Supplementary Material**.

### 3.1 Correlations with clinical symptoms

DT to faces in all the pictures correlated negatively with the communication (r=–0.32; p<0.01) and repetitive stereotyped behaviour sub-scores (r=–0.27; p<0.05) on the SCQ but the correlation with the social reciprocal interaction sub-score was not significant (r=–0.09; p>0.05). DT to faces did not significantly correlate with the ADOS emotion recognition subscore or Conners’ IA or HI T-score (p>0.05).

### 3.2 Hierarchical regression

For ADOS emotion recognition subscore and eye tracking variables analysed in the pictures, neither IQ nor oppositional symptoms explained a significant amount of the variance over and above ASD or ADHD factors. Hierarchical linear regression model results tables for each dependent variable are presented in the **Supplementary Material**.

## 4 Discussion

In the present study, we assessed visual attention to ADOS pictures amongst children and adolescents with ADHD, ASD, ADHD+ASD, and Neurotypicals. We found slower orientation to ADOS pictures, indexed by FFT (First Fixation Time), and reduced viewing time, indexed by DT (Dwell Time), in those with ASD (ASD and ASD+ADHD) compared to those without ASD (controls and ADHD). We did not find a significant main effect of ADHD on visual attention or a significant ASD*ADHD interaction. As summarised in **Table 2**, these findings suggest that those with ASD (ASD and ASD+ADHD) have reduced visual attention to ADOS pictures compared to those without ASD (ADHD and Neurotypical controls).

As shown in **Figure 1**, There was a significant interaction of ASD factor by Interest Area (Faces versus Non-social areas). When this was followed up by simple effects analysis, DT to faces was reduced in those with ASD (ASD and ASD+ADHD) compared to those without ASD (ADHD and Neurotypical controls). Furthermore, DT to faces in pictures was associated with symptom severity on the SCQ (ASD) but not the Conners’ IA or HI subscales (ADHD). These findings are suggestive of an association of atypical visual social attention with ASD but not ADHD symptom levels and, confirm the factorial analysis findings that ASD is driving the influence of reduced visual attention to faces in ADOS pictures.

Findings of slower orientation, indexed by FFT and reduced viewing time, indexed by DT to faces (social interest areas) in ADOS pictures in children with ASD (ASD and ASD+ADHD) is in line with the eye tracking literature on social scene perception in ASD (25, 26, 46). EEG studies have also found atypical ERP responses to faces in those with ASD (ASD and ASD+ADHD), supporting face processing deficits in ASD at the neural level (44, 47). Our findings from ADOS pictures suggest that the ASD+ADHD group genuinely have visual social-emotional attention like ASD rather than a ‘phenocopy’ or a severe form of ADHD as, we found a significant main effect of ASD but did not find a significant main effect of ADHD or a significant ASD*ADHD interaction.

Spontaneous emotion recognition was measured using the ADOS emotion recognition subscore. Children with ADHD had increased scores on the ADOS emotion recognition subscore which were not significantly different from those with ASD or ASD+ADHD. Although ADOS was not completed in typically developing children, if we consider that an ADOS emotion recognition score of 0 would be the expected score in typically developing children, then those with ADHD have raised scores. Thus, all clinical groups showed raised scores on the ADOS emotion recognition subscore. As predicted, the ASD group had the highest scores, suggestive of more difficulties with spontaneous emotion recognition (although this was not statistically significant amongst the clinical groups; **Table 1**).

Children with ADHD without comorbid ASD showed normal visual attention to ADOS pictures, which were predominantly of positive emotional valence. Our findings are in keeping with Serrano et al. (2018) who found highest effect sizes for reduced visual attention to angry (d=-0.73) and scared faces (d=-0.50) in social scenes in children with ADHD (27). Pishyareh et al. (2015), however, found reduced fixation latencies to pleasant versus unpleasant pictures, in children with ADHD, which differs from our findings albeit with different fixation latencies and methodology as they presented pictures stimuli side by side and did not capture attention to faces (48). Additionally, differences between studies could also be explained by the inclusion of children with oppositional symptoms in our study. Indeed, this association between social communication difficulties and conduct problems including oppositional behaviours in children with ADHD has been found in larger samples previously (8, 49, 50). Furthermore, Santosh et al. (2004) also found an association with ADHD, social communication difficulties and conduct problems with relational difficulties with peers on parental report (49). We did not find an association with visual attention and Conduct problems in this study, however as we did not include pictures of negative valence this could explain the difference in findings.

We did not find an association with visual attention and IQ in this study. This could be because of the free viewing task of shorter duration in this study, requiring minimal cognitive effort compared to longer emotion recognition tasks (27).

In this study, a dissociation was found with reduced viewing time to faces in those with ASD (ASD and ASD+ADHD) but not ADHD (without ASD); suggesting reduced interest to faces is a specific finding in those with ASD.Furthermore, viewing time to faces, indexed by Dwell Time (DT) was significantly negatively correlated with communication and repetitive stereotyped behaviour severity on the SCQ. This would suggest that reduced viewing time to faces in ADOS pictures is associated with poorer communication and more restrictive and repetitive behaviours in our study. DT to faces was not significantly associated with the social reciprocal interaction sub-score on the SCQ and as discussed previously, children with ADHD have difficulties with social interaction. Our findings therefore support communication and repetitive stereotyped behaviour SCQ subscale interpretation when assessing for ASD in ADHD.

It could be postulated that aspects of visual social attention that are thought to be more classical ASD deficits seem to be preserved in ADHD. For example, preserved visual social-emotional attention to faces in ADOS pictures compared to those with ASD. It could be postulated that the ADOS pictures were rewarding and motivating to look at as they were colourful with no explicit task requiring sustained attention. As motivation and reward have been shown to be as effective as stimulant medication for those with ADHD (51), preserved visual social attention to ADOS pictures without expense to other areas of visual attention could be quite likely. Furthermore, our findings could suggest that social impairments and atypical visual social-emotional attention in ADHD may not be pervasive but rather context dependent and potentially influenced by intensity of stimulation, arousal, emotional valence, and reward (52).

### 4.1 Limitations, strengths and future directions

Due to study time limitations, participants in this sample were subject to a small number of trials (n=3). However, this allowed for more bottom-up processing and increased saliency with less habituation. The sample size (n=84) is not big enough to look at subsamples (e.g., girls). Our findings are therefore potentially less generalisable to the whole neurodevelopmental population.

We looked at positive valence images in this study; however, comparing this with negative valence pictures could also be helpful. Future avenues, such as incorporating the pupil as a measure of arousal and measures of peer relations or emotional liability, could help to uncover further insights into atypical visual social attention in ASD and ADHD.

Despite the small sample size, the participants were well categorised and all children with ADHD were either stimulant naive or taken off stimulant medication minimising confound due to medication. Further studies would benefit from taking this multisite approach so that analysis of subsamples such as within the comorbid group and in girls is more feasible.

The DAWBA was used as opposed to more lengthy semi-structured interviews such as the ADI-R to aid the categorisation of ASD. The SCQ, however, relates closely to the ADI-R and the use of the SCQ in combination with the DAWBA has been used by the assessors (PK and CH) in a large longitudinal study previously (31, 32).

Due to study time limitations, the ADOS was not completed for the typically developing group, meaning that comparisons for ADOS scores including the emotion recognition subscore could only be made amongst the clinical groups. As an ADOS score of 0 is denoted as typically developing, this can be substituted, allowing for a ‘pseudo’ comparison amongst the groups, but it is acknowledged that this was not explicitly tested and therefore was not carried out in the analysis.

In terms of study design, it is also important to consider the limitations of eye tracking. Firstly, there are technical issues with eye tracking. Head movements, eye blinking and participant fatigue can lead to artefact data which requires data cleansing. Eye tracking measures are sensitive to luminance, image properties such as contrast and spatial properties. Although luminance was measured and kept constant, it was not possible to control for some of the other stated factors as images were not manipulated. There are potential confounding factors when studying visual attention using eye tracking, for example lighting and cognitive loading of the task. Visual attention in the context of this study explored overt attention in relation to active vision (53). This design does not take covert attention into consideration thereby potentially missing the effects of early visual attentional processes that neural EEG techniques would detect.

A 2x2 factorial approach to examine the effects of ADHD and ASD as fixed factors on visual social-emotional attention variables was used as it is commonly used in studies of comorbidity (43, 44, 54). The 2x2 factorial approach has the ability to differentiate the effect of ADHD, ASD and of comorbidity through an interaction of fixed factors allowing for the research questions to be tested. Furthermore, it improves the power of the sample by the combination of 2 groups into one factor, essentially doubling the power of a more traditional group effect. Interpretation and comparison of findings (e.g. pure group versus controls) can be more difficult in the 2x2 factorial approach compared to the group approach. This was less of a problem for this study, as the main question was regarding ADHD versus ASD and comorbidity, however raw eye tracking data in the groups is provided in the **Supplementary Materials**.

Hierarchical linear regression was used to test for added variance of IQ and oppositional symptoms over and above ADHD and ASD on the significant eye tracking variables as clinical groups differed on IQ and oppositional symptoms have been shown to be important in social problems in ADHD (49). Although, it could have been argued that analysis of covariates (ANCOVA) could have been used, the use of ANCOVA has been shown to be a poor way of covarying for IQ in psychiatric and developmental disorders especially if IQ is tightly bound to the clinical profile of the disorder as is the case for both ASD and ADHD (55). Miller and Chapman (2001) proposed that having a control group with a lower IQ would be the best way to examine the effects of IQ. Unfortunately, this was not possible in our studies due to recruitment difficulties and time limitations, but future study designs will benefit from controls with lower IQ (55). It would also be important to consider comparison of groups with other psychiatric disorders as studies have shown that visual attention can be affected in conduct and emotional disorders at the diagnostic level (56, 57).

### 4.2 Clinical Practice Implications

The ADOS was originally designed as a research tool in the assessment of ASD and has become commonly adopted in clinical practice as a tool to standardise observed autistic behaviours as part of the diagnostic process (58, 59). As the clinical phenotype of ASD has broadened, there has been suggestion that the ADOS may not be sensitive enough to pick up all of these cases and can also be prone to assessor subjectivity (14). Furthermore, the ADOS was not originally designed to be used in clinically complex cases with high levels of comorbidity. Although the ADOS is an observer-based tool by a trained clinician, it can still be prone to subjective bias by the administrator. Our findings however seemed to validate the use of the ADOS; when children viewed ADOS pictures, visual attention to faces was reduced in those with ASD compared to those without. These findings are in line with findings in the literature using complex and dynamic scenes (19). Those with ADHD (without ASD) did not show these atypicalities to ADOS pictures; in particular, they did not show slower orienting to ADOS pictures or reduced viewing time to faces.

We found that atypical visual attention to ADOS pictures is an indicator of ASD symptoms. Measuring atypical visual social attention could be a helpful adjunct to ADOS examination when assessing for neurodevelopmental conditions with social cognitive deficits. Furthermore, visual social attention measurement may have a role in those cases that are missed by standard clinical assessments or in cases where there is controversy, or a second opinion is being sought.

Findings suggest that atypical visual social attention to ADOS images could be a potential utility for differentiating the groups. Prediction of diagnoses from significant atypical visual social-emotional attention measures could be tested in future studies on a larger scale.

It is interesting to note that there were more children in the ASD+ADHD group than the ADHD group in this study. As these samples are from the clinic population, this is in keeping with comorbidity being common in child psychiatry (60). As we found that those with ASD+ADHD had atypical visual attention to ADOS pictures, associated with ASD symptom severity, our findings support the early assessment of comorbid ASD in ADHD. Unfortunately, this is often not the case in clinical practice (61, 62). Prompt assessment of comorbidity would allow for timely treatment approaches for additional socio-emotional difficulties in children with ASD+ADHD, with the potential to improve long term outcome.

## 5 Conclusions

In conclusion, we found that visual attention to faces was reduced in those with ASD (ASD and ASD+ADHD) compared to those without ASD (ADHD and NT). Reduced visual attention to faces in the whole sample was associated with Autism symptom severity (SCQ subscale scores) but not ADHD symptom severity (CRS-3 scores). Our findings provide preliminary evidence in support of implementing visual attention measurement during assessment of ASD in the context of comorbidity with ADHD. For example, if a child with ADHD was found to reduce attention to faces in ADOS pictures this may suggest additive difficulties on the autism spectrum. Replication across a larger sample would be informative. This work has future potential in the clinic to help with complex cases, including those with co-occurring ADHD and ASD.

## Data availability statement

The original contributions presented in the study are included in the article/**Supplementary Material**, further inquiries can be directed to the corresponding author/s.

## Ethics statement

The studies involving humans were approved by East Midlands Research Ethics Committee (REC) (17/EM/0193), IRAS project number 220158. The studies were conducted in accordance with the local legislation and institutional requirements. Written informed consent for participation in this study was provided by the participants’ legal guardians/next of kin.

## Author contributions

PK: Conceptualization, Data curation, Formal analysis, Investigation, Methodology, Project administration, Software, Writing – original draft, Writing – review & editing. IA: Data curation, Project administration, Software, Writing – review & editing. AB: Data curation, Writing – review & editing. DR: Funding acquisition, Writing – review & editing. CH: Conceptualization, Resources, Supervision, Writing – review & editing, Funding acquisition. MG: Conceptualization, Formal analysis, Funding acquisition, Resources, Supervision, Writing – review & editing, Investigation, Methodology, Validation.
