# Supplementary material for: A comparison of visual attention to pictures in the Autism Diagnostic Observation Schedule in children and adolescents with ADHD and/or autism
Source: Front Psychiatry. 2024 Apr 29;15:1378593. doi: 10.3389/fpsyt.2024.1378593 (PMC11089217; doi:10.3389/fpsyt.2024.1378593)
Supplement: Supplementary file 1 [file Table_1.docx]

# Supplementary Materials

# ADOS emotion recognition subscore (further information)

The ADOS emotion recognition score refers to the subscore B4 and B5 in ADOS modules 3 and 4 respectively. This subscore is termed comments on others’ emotions/empathy. The scoring details and wording of this item are taken directly out of the ADOS-2 module 3 and 4 booklet and can also be found in the ADOS-2 manual (40).

*“The focus of this item is on the participants’ communication of his or her recognition, understanding, and/or response to the feelings of other people or characters, real or conveyed in stories or other tasks.*

*“0=Spontaneously communicates clear understanding or labelling of and/or appropriate response to several different emotions in other people/characters. Labelling several emotions in others is sufficient but not necessary if there are other clear indications of understanding and/or appropriate response.*

*“1=Communicates some understanding, labelling, or response to an emotion in others (e.g. spontaneously and correctly identifies at least one emotion in another person/character).*

*“2=No or minimal identification/communication of understanding of emotion in others.”*

# Supplementary Table

Significant multivariate effects of Interest Area, Picture and Picture by Interest Area on all the eye tracking variables that were independent of the fixed factors (ASD factor and ADHD factor) are tabulated below (Table S.1)

Table S.1: Summary of significant main effects independent of fixed factors in ADOS pictures for all children

| **Dependent variable (s)** | **Independent variable/**  **Interaction** | **Main effect (F)** | **Direction of effect^b^**  **(mean dependent variable)** |
| --- | --- | --- | --- |
| **Multivariate tests on all eye tracking variables (DT, FC, FFT)** | Interest Area | 20.90^c^ | - |
|  | Picture | 8.92^c^ | - |
|  | Interest Area*Picture | 5.97^c^ | - |
| **Planned contrasts from univariate tests^a^** |  |  |  |
| **DT (ms)** | Interest Area | 26.83^c^ | Faces (2562.40) > non-social (1594.61)^c^ |
| **FC** | Interest Area | 23.30^c^ | Faces (8.67) > non-social (5.75)^c^ |
| **FFT (ms)** | Interest Area | 8.63^c^ | Faces (3671.62) < non-social (4922.76)^d^ |
| **DT (ms)** | Picture | 50.45^c^ | Meal (1305.14) < Hollywood (2848.55)^c^ > holiday (2081.83)^c^ |
| **FC** | Picture | 51.97^c^ | Meal (4.55) < Hollywood (10.0)^c^ > holiday (7.08)^c^ |
| **FFT (ms)** | Picture | 16.64^c^ | Meal (5900.03) > Hollywood (3264.43) ^c^ < holiday (3727.11) |
| **DT (ms)** | Interest Area*Picture | 0.01 | - |
| **FC** | Interest Area*Picture | 0.10 | - |
| **FFT (ms)** | Interest Area*Picture | 9.18^d^ | Faces (3804.66) < non-social (7995.40) in holiday^c^  Faces (3158.73) < non-social (4295.49) in meal^e^  Faces (4051.47) > non-social (2477.39) in Hollywood^e^ |

^a^Planned contrasts were conducted on significant univariate tests from the multivariate analysis. For repeated planned contrasts, each category (except the first) is compared to the previous category.

^b^Significant univariate main effects from planned contrasts and significant interactions were followed up by pairwise comparisons (LSD) from simple effects analysis

^c^p<0.001; ^d^p<0.01; ^e^p<0.05. DT (Dwell Time); FC (Fixation Count); FFT (First Fixation Time); ms (milliseconds)

# Supplementary data from hierarchical regression

Hierarchical linear regression model tables described in the results section were presented here (Tables S.2-S.5). For each model the p-value was denoted as significant if p<0.05. If the p-value for a model was between 0.05–0.10, this value was shown in the table to allow for trends in the data to be presented. Significant *R^2^* change within each model was denoted as p<0.05.

**Table S.2: Hierarchical linear regression models for ADOS emotion recognition score^†^**

| **Model** (predictors) | ***R^2^*** | ***R^2^* change** | ***F*** | **p-value** |
| --- | --- | --- | --- | --- |
| **1**  (ASD factor) | 0.06 | 0.06 | 3.70 | =0.06 |
| **2**  (ASD factor, ADHD factor) | 0.06 | 0.00 | 1.90 | n/s |
| **3**  (ASD factor, ADHD factor, FSIQ) | 0.06 | 0.00 | 1.22 | n/s |
| **4**  (ASD factor, ADHD factor, FSIQ Conners’ OD T score) | 0.07 | 0.00 | 0.93 | n/s |
|  |  |  |  |  |

**^†^**ADOS score only available for clinical cases. FSIQ (Full Scale Intelligence Quotient); OD (Oppositional); *(significant *R****^2^*** change; p<0.05); n/s (non-significant)

**Table S.3: Hierarchical linear regression models for DT to faces in ADOS pictures**

| **Model** (predictors) | ***R^2^*** | ***R^2^* change** | ***F*** | **p-value** |
| --- | --- | --- | --- | --- |
| **1**  (ASD factor) | 0.06 | 0.06* | 4.70 | <0.05 |
| **2**  (ASD factor, ADHD factor) | 0.06 | 0.00 | 2.33 | n/s |
| **3**  (ASD factor, ADHD factor, FSIQ) | 0.06 | 0.00 | 1.59 | n/s |
| **4**  (ASD factor, ADHD factor, FSIQ Conners’ OD T score) | 0.06 | 0.00 | 1.18 | n/s |
|  |  |  |  |  |

FSIQ (Full Scale Intelligence Quotient); OD (Oppositional); *(significant *R****^2^*** change; p<0.05); n/s (non-significant)

**Table S.4 Hierarchical linear regression models for FC to faces in ADOS pictures**

| **Model** (predictors) | ***R^2^*** | ***R^2^* change** | ***F*** | **p-value** |
| --- | --- | --- | --- | --- |
| **1**  (ASD factor) | 0.04 | 0.04 | 3.52 | =0.06 |
| **2**  (ASD factor, ADHD factor) | 0.05 | 0.00 | 1.90 | n/s |
| **3**  (ASD factor, ADHD factor, FSIQ) | 0.05 | 0.00 | 1.30 | n/s |
| **4**  (ASD factor, ADHD factor, FSIQ Conners’ OD T score) | 0.05 | 0.00 | 0.96 | n/s |
|  |  |  |  |  |

FSIQ (Full Scale Intelligence Quotient); OD (Oppositional); *(significant *R****^2^*** change; p<0.05); n/s (non-significant)

**Table S.5: Hierarchical linear regression models for FFT to faces in ADOS pictures**

| **Model** (predictors) | ***R^2^*** | ***R^2^* change** | ***F*** | **p-value** |
| --- | --- | --- | --- | --- |
| **1**  (ASD factor) | 0.08 | 0.08* | 6.32 | <0.05 |
| **2**  (ASD factor, ADHD factor) | 0.08 | 0.00 | 3.31 | <0.05 |
| **3**  (ASD factor, ADHD factor, FSIQ) | 0.09 | 0.02 | 2.64 | =0.06 |
| **4**  (ASD factor, ADHD factor, FSIQ Conners’ OD T score) | 0.10 | 0.00 | 2.07 | =0.09 |
|  |  |  |  |  |

FSIQ (Full Scale Intelligence Quotient); OD (Oppositional); *(significant *R****^2^*** change; p<0.05); n/s (non-significant)

**Supplementary raw eye tracking data in the groups**

Raw eye tracking data in the groups are presented in Table S.6.

**Table S.6: Raw eye tracking data in the groups**

| **Mean** | **Group** | | | |
| --- | --- | --- | --- | --- |
|  | **ADHD**  (n=16) | **ASD**  (n=18) | **ASD+ADHD**  (n=28) | **Controls**  (n=22) |
| **DT to faces**  (SD; range) | **2614.63**  (1159.19;  565.33-4207.33) | **1983.69**  (1154.96;  568.00-4486.00) | **2425.38**  (1582.36;  683.33-6926.00) | **3225.91**  (1331.61;  1094.0-5944.67) |
| **FC to faces**  (SD; range) | **8.42**  (4.54;  1.67-16.67) | **7.08**  (4.72;  1.00-18.67) | **8.17**  (4.88;  2.00-22.67) | **11.02**  (4.48;  3.67-21.67) |
| **FFT to faces**  (SD; range) | **3105.42**  (3583.92;  38.67-13871.33) | **5415.84**  (3615.02;  918.00-10568.67) | **3900.94**  (3097.32;  38.67-11026.12) | **2264.27**  (1227.13;  807.33-5193.33) |

DT (Dwell Time); FC (Fixation Count); FFT (First Fixation Time)
